# High-Silica Layer-like Zeolites Y from Seeding-Free Synthesis and Their Catalytic Performance in Low-Density Polyethylene Cracking

**DOI:** 10.1021/acsami.1c21471

**Published:** 2022-01-25

**Authors:** Bastian Reiprich, Karolina A. Tarach, Kamila Pyra, Gabriela Grzybek, Kinga Góra-Marek

**Affiliations:** Faculty of Chemistry, Jagiellonian University in Kraków, Gronostajowa 2, 30-387 Kraków, Poland

**Keywords:** layer-like zeolites, faujasite, operando spectroscopy, LDPE cracking, TPO coke studies

## Abstract

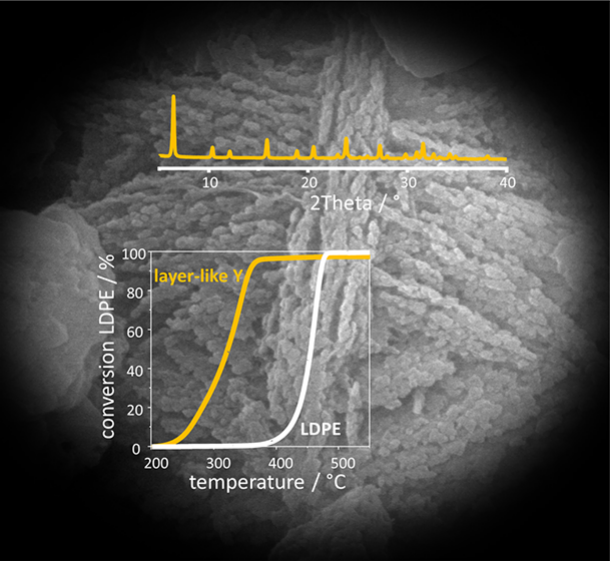

Layer-like FAU-type zeolite Y was synthesized by an organosilane-assisted low-temperature hydrothermal method and its catalytic activity was verified in the low-density polyethylene (LDPE) cracking process. The synthesis procedure of high-silica layer-like zeolite Y was based on organosilane as a growth modifier, and for the first time, the seeding step was successfully avoided. The X-ray diffraction and electron microscopy studies, scanning electron microscopy and transmission electron microscopy confirmed the formation of pure FAU structure and zeolite particles of plate-like morphology arranged in the manner of the skeleton of a cuboctahedron. The *in situ* Fourier transform infrared (FT-IR) spectroscopic studies, low-temperature nitrogen sorption, and electron microscopy results provided detailed information on the obtained layer-like zeolite Y. The acidic and textural properties of layer-like zeolites Y were faced with the catalytic activity and selectivity in the cracking of LDPE. The quantitative assessment of catalyst selectivity performed in FT-IR/GC–MS *operando* studies pointed out that LDPE cracking over the layer-like material yielded value-added C_3_–C_4_ gases and C_5_–C_6_ liquid fraction at the expense of C_7+_ fraction. The detailed analysis of coke residue on the catalyst was also performed by means of FT-IR spectroscopy, thermogravimetric analysis, and thermoprogrammed oxidation coupled with mass spectrometry for the detection of oxidation products. The acidic and textural properties gave a foundation for the catalytic performance and coking of catalysts.

## 1 Introduction

Many important large-scale chemical processes such as fluid catalytic cracking or hydrocracking use faujasite (FAU) zeolites as the catalyst or support material, respectively. This type of zeolites gained their importance in the chemical industry by beneficial textural properties, such as a three-dimensional pore geometry, a pore window diameter of 0.74 nm, and a super-cage diameter of 1.12 nm, which makes them belonging to large-pore zeolites. Also, their chemical properties, such as a variable Si/Al molar ratio from 1 (as-synthesized)^1^ up to a highly or even pure siliceous material (by dealumination)^2^ and a high hydrothermal stability are beneficial. Faujasite as a catalyst in the H-form with acid sites has to be obtained with the molar Si/Al ratio higher than 1.5, and then, it is called a zeolite Y.^3^ Also, an increased hydrothermal stability is found at higher molar Si/Al ratios. The usual way to achieve Si/Al molar ratios higher than 1.5 is the preparation of two synthesis gels: a seed gel and a feedstock. In this case, the seed gel usually undertakes a 1-day aging step at an ambient temperature to form seed crystals. The addition of the seed gel to the feedstock gel accelerates the crystal growth. This technique is commonly known and results in high-quality zeolite Y samples. However, the disadvantage is the necessity of an additional preparation step. An elegant route to avoid it was investigated by Liu et al. by adding sulfuric acid to the synthesis gel and thus reducing the basicity and obtaining high-silica zeolite Y.^4^ Still, zeolites with a conventional purely microporous texture have a disadvantage of the limited diffusion of reactants within the pore system, which can cause lower catalytic activity, selectivity, or even coking and deactivation of the zeolite. To overcome a reduced diffusion of molecules within the zeolitic framework, the creation of an additional pore system (meso- and/or macropores) can be a proper solution. An additional pore system can be created by applying top-down or bottom-up strategies. In a top-down route, an already synthesized zeolite is treated with acid, base, and/or steam to remove framework atoms. In a bottom-up route, the zeolite synthesis is modified by additives or adjusting, for instance, the temperature to achieve an additional pore system. An additional pore system within the framework of an FAU-type zeolite can be introduced in the bottom-up route by adding an organosilane to the synthesis gel and obtaining zeolite crystals with a layer-like morphology. The organosilane acts in this case as a growth modifier as well as a mesoporogen. This procedure was first applied for FAU-type zeolite X by Inayat et al. in 2012.^5^ Later on, the growth mechanism and catalytic activity of layer-like zeolite X were investigated.^6−12^ The layer-like morphology which is not in the nature of a faujasite zeolite, usually an octahedral morphology occurs, is due to the presence of small amounts of FAU/EMT intergrowth within the dominant FAU phase.^13,14^ This is causing the layer-like growth and the branching of plates, thus creating a hierarchical morphology. To apply layer-like FAU-type materials for acid-catalyzed reactions such as catalytic cracking, the Si/Al molar ratio has to be increased to obtain a zeolite Y material. Many research groups carried out investigations on the synthesis of layer-like zeolite Y using an organosilane as the growth modifier, and all of them applied a synthesis related to the conventional procedure for zeolite Y material, namely, the application of seeding by working with a seed gel and a feedstock gel or by just adding zeolite Y seed crystals to the synthesis mixture.^15−21^

Here, we report for the first time a synthesis of high-silica layer-like zeolite Y using an organosilane as a growth modifier but without the application of any seeding. To obtain these materials, the synthesis concept from Liu et al.^4^ using sulfuric acid to reduce the basicity of the synthesis gel was adapted and modified. The high-silica zeolite Y was found to be a very effective catalyst in polypropylene cracking, providing high selectively to C_4_–C_9_ hydrocarbons as resulting products.^22^ A mesoporous zeolite USY prepared by a sequence of processes with desilication as final one offered an enhanced low-density polyethylene (LDPE)-cracking efficiency by means of the lowering of the cracking temperature.^23^ Similarly, the HUSY zeolites with various unit cell sizes (CBV760, CBV712, and CBV500) in tertiary recycling of polypropylene by catalytic cracking in a semibatch stirred reactor have proved to be effective catalysts.^24,25^ It has been shown that neither the concentration nor the strength of the acid sites is the most important factor for the cracking of plastic waste. The determinant of plastic waste cracking has been identified as the secondary mesoporous structure.^25^ Thus, it was of our interest if the bottom-up modified layer-like zeolites Y can offer high catalytic activity in LDPE cracking as materials of hierarchical macro-/mesoporous structure.

## 2 Experimental Section

### 2.1 Materials

Layer-like FAU-type zeolite Y was synthesized by a modified hydrothermal synthesis for conventional zeolite Y from Liu et al.^4^ The main differences were the addition of an organosilane acting as a template/growth modifier and a reduced crystallization temperature. Sodium hydroxide (98.9%, Honeywell) as well as sodium aluminate (54.0% Al_2_O_3_, 42.0% Na_2_O, Sigma-Aldrich) were both mixed separately with deionized water, and the solutions were cooled down to room temperature. The sodium hydroxide solution and the sodium aluminate solution were mixed in a PP bottle at 300 rpm for 5 min using a two-bladed centrifugal stirrer. LUDOX AS40 (39.9% SiO_2_, Sigma-Aldrich) was added slowly while stirring at 1300 rpm. Sulfuric acid (96.3%, Merck) was added dropwise to the synthesis mixture while stirring, followed by agitation for 1 h. The organosilane 3-(trimethoxysilyl)propyl octadecyl dimethyl ammonium chloride (TPOAC, 42 wt % in methanol, Sigma-Aldrich) was added dropwise, followed by stirring for 30 min at 1300 rpm. The obtained synthesis gels had a molar composition of Al_2_O_3_:4Na_2_O:3SiO_2_:180H_2_O: 0.67H_2_SO_4_:*a*TPOAC, with *a* = 0.144, 0.225. The synthesis gel was kept under static conditions at room temperature for 1 day, which is considered the aging step. The crystallization was carried out under static conditions in a furnace at 75 °C for 5–10 days. As the synthesis should be kept simple, the temperature was, compared to the synthesis from Liu et al.,^4^ reduced from 100 to 75 °C and PP bottles were used instead of stainless-steel autoclaves. The zeolite product was separated from the suspension using a Büchner funnel, washed with deionized water up to a pH value of 8, and dried in a furnace at 75 °C overnight. The organic template was removed by calcination in a muffle furnace for 8 h at 550 °C (2 °C·min^–1^).

For comparison, a conventional zeolite Y was synthesized by a similar synthesis procedure but in the absence of organosilane. The obtained synthesis gel had a molar composition of Al_2_O_3_:4Na_2_O:3SiO_2_:180H_2_O:0.67H_2_SO_4_. After the aging step under static conditions at room temperature, the crystallization was carried out under static conditions in a furnace at 75 °C for 5 days. A calcination step was not carried out.

The obtained layer-like zeolite Y samples were in the Na^+^-form after the synthesis. For further application as catalysts, an ion-exchange with NH_4_^+^-ions was carried out followed by a calcination step to obtain the H-form. The ion-exchange was carried out in a PP bottle at 60 °C (oil bath) for 6 h using a 1 molar aqueous ammonium nitrate solution (ammonium nitrate: 98%, Merck). The zeolite: water mass ratio was 1:30. The suspension was stirred at 500 rpm using a magnetic stirring bar. Afterward, the zeolite was separated from the suspension by centrifugation (5 min at 9000 rpm). The centrifugation step was repeated several times by removing the clear supernatant and adding deionized water to wash out excess ions (total wash water/suspension = 10:1). The recovered zeolite product was dried at 75 °C overnight and the ion-exchange was repeated two more times. After three ion-exchange steps, the NH_4_–zeolites were calcined in a muffle furnace at 500 °C for 2 h (2 °C·min^–1^) to obtain H-zeolites.

The obtained layer-like and conventional zeolite Y samples, respectively, were designated as LY-a for layer-like faujasites, where “a” stands for the TPOAC/Al_2_O_3_ molar ratio of the synthesis gel composition, and “CY” for conventional faujasite. For zeolite samples after ion-exchange (NH_4_^+^-form) and calcination (H-form), the suffixes −NH_4_ and −H, respectively, are added, for example, LY-a, LY-a-NH_4_, and LY-a-H.

For comparison in the catalytic testing, commercial zeolite Y samples CBV100 (Si/Al = 2.73, Na-form) and CBV760 (Si/Al = 30, H-form) were purchased from Zeolyst International. For CBV100, an ion-exchange and a calcination step to obtain the H-form were carried out (denoted as CBV100-H). CBV760 is denoted as CBV760-H, as it was supplied in the H-form.

### 2.2 Characterization Methods

The crystal structure and relative crystallinity were determined by powder X-ray diffraction (XRD) patterns obtained from a Rigaku Multiflex diffractometer equipped with Cu Kα radiation (40 kV, 40 mA). The scanning range was of 3–50° 2θ, with a scan speed of 2 deg·min^–1^. The chemical composition, namely, the Si/Al molar ratios, of the zeolite samples were analyzed by inductively coupled plasma optical emission spectrometry (ICP–OES, Optima 2100DV, PerkinElmer). The textural properties were measured by low-temperature physisorption of nitrogen using a Quantachrome Autosorb-1-MP gas sorption analyzer. Prior to exposure to nitrogen at −196 °C, the zeolite samples were dehydrated at 350 °C for 24 h under high vacuum conditions (ca. 10^–5^ mbar). The following methodologies were applied to obtain textural properties: specific surface area (*S*_BET_) by the Brunauer–Emmet–Teller method, specific external surface area (*S*_ext_) by the *t*-plot method, micropore volume (*V*_micro_) by the *t*-plot method, and total pore volume (*V*_tot_) by single point adsorption at p·p_0_^–1^ = 0.984.

The scanning electron microscopy (SEM) micrographs were obtained using a FEI Quanta 3D FEG microscope. Prior to measurement, the materials were coated with a Pd/Au layer.

Prior to the Fourier transform infrared (FT-IR) study, the zeolite was pressed into a self-supporting wafer (ca. 10 mg·cm^–2^) and pretreated *in situ* in a quartz IR cell at 450 °C under vacuum conditions (10^–5^ mbar) for 1 h. The spectra were recorded with a resolution of 2 cm^–1^ in a Bruker Vertex 70 spectrometer equipped with an MCT detector. All spectra presented in this study were normalized to 10 mg of sample what corresponded also the same intensities of overtone bands (1800–1400 cm^–1^). The sorption of CO (PRAXAIR, 9.5) was performed at −120 °C up to maximum intensities of the bands at 2230–2190 cm^–1^, that is, up to the total saturation of the Lewis acid sites. The total concentrations of acid sites, both Brønsted and Lewis type, were determined by quantitative IR studies of ammonia (NH_3_) sorption (PRAXAIR, 3.5).^26^ The amount of NH_3_ sufficient to neutralize all acid sites was adsorbed at 200 °C under static conditions. Subsequently, the gaseous and physisorbed ammonia molecules were evacuated for 20 min under vacuum at the same temperature, which was documented as the disappearance of the bands related to gaseous and physisorbed NH_3_ in the collected spectrum. The band intensities in the latter spectrum were used to calculate the total concentration of Brønsted and Lewis sites using the intensities of the 1450 cm^–1^ band of ammonium ions (NH_4_^+^) and the 1620 cm^–1^ band of NH_3_ coordinatively bonded to Lewis sites (NH_3_L). The following absorption coefficients were applied: 0.11 cm^2^·μmol^–1^ for the ammonium ion band (NH_4_^+^) and 0.026 cm^2^·μmol^–1^ for the 1620 cm^–1^ band of ammonia ligated to Lewis sites (NH_3_L).^26,27^ The strength of the acid sites was derived from ammonia thermodesorption FT-IR studies designated as the ratio of the adsorbed amount of NH_3_ at 350 °C compared to the adsorption at 200 °C.

The concentrations of the Brønsted acid sites accessible for bulky 2,6-di*tert*-butylpyridine (di-TBPy, Sigma-Aldrich, >97%) were obtained from experiments where excess of di-TBPy was adsorbed at 200 °C. The physisorbed molecules were desorbed under vacuum at the same temperature and the sample was cooled to room temperature. The 1615 cm^–1^ band intensities of di-TBPyH^+^ with absorption coefficient 0.50 cm^2^·μmol^–1^ were used to quantify the number of di-TBPy-accessible acid sites.^28^ The maximum intensity of di-TBPyH^+^ band was calculated from the spectra recorded upon the heating of zeolite contacted with di-TBPy vapor at 200 °C for 15 min, and subsequently cooled down to RT.

### 2.3 Catalytic Tests of LDPE Cracking

For the catalytic tests, LDPE (Alfa Aesar, product no.: 42607, lot no.: P28D047) was crushed, powdered, and sieved (250 μm). The catalytic cracking of LDPE was assessed by thermogravimetric analysis (TGA) using a TGA/SDTA Mettler Toledo apparatus. The zeolite powder (10 mg) together with polymer (30 mg) were mixed (10 min) in an agate mortar and then ca. 10 mg of the prepared mixture was transferred to a α-Al_2_O_3_ crucible and weighted with a Mettler Toledo balance before the analysis. Decomposition of the polymer was carried out in a temperature range from 30 to 600 °C at the heating rate of 5 °C min^–1^ under a N_2_ flow (80 mL·min^–1^). In the conversion calculations, the catalyst weight and adsorbed moisture content were considered. In addition, the polymer was cracked without the aluminosilicate catalyst addition for comparative purposes. The coke content was calculated from further TGA experiments. During polymer cracking, the sample after heating to 600 °C was subjected to the flow of synthetic air (80 mL·min^–1^) and with a rate 30 °C·min^–1^ heated to 800 °C until no mass change was observed.

### 2.4 FT-IR *Operando* Catalytic Studies

The *operando* system connected to a flow setup was used to investigate the degradation of polyethylene (LDPE). For this purpose, in a custom-made 2 cm^3^ volume quartz IR cell, the self-supporting disc was placed (ca. 5.5–6 mg·cm^–2^) consisting of the catalyst and LDPE mixed in 1:1 ratio. The custom-made spectroscopic cell is delivered by MeasLine (www.measline.com) company under a licensed patent (PL232633, Poland). The homogeneity of the zeolite/catalyst mixture used for TGA and *operando* purposes was followed by the comparison of their IR spectra recorded at room temperature and normalized to the same intensity of the overtone bands (2050–1800 cm^–1^). Each catalyst was reflected in the same intensity of the bands 2960 cm^–1^ (−CH_3_ group) and 2925 cm^–1^ (−CH_2_ group), thus the equal proportion of zeolite and LDPE in all samples. Under the reaction conditions, the catalyst surface as well as gas phase were simultaneously monitored. As a carrier gas, nitrogen (30 mL·min^−1^) introduced by Teflon lines (1/16″) was used, kept at 110 °C. The *operando* IR cell with placed catalyst disc was rapidly heated from room temperature up to 220 °C with a ramping rate of 10 °C/s. The test was performed at 220 °C until the polymer entire decomposition was achieved. Time-resolved spectra were recorded on a FT-IR spectrometer Vertex 70 (Bruker) equipped MCT detector with the spectral resolution of 2 cm^–1^ and the 80 kHz scanner velocity. In parallel to spectroscopic observations, the reaction products were simultaneously analyzed by mass spectrometry (MeasLine, www.measline.com, RGA200) as well as gas chromatography (Agilent Technologies 7890B). In the selectivity of the catalysts, the resulting coke and tar were considered.

## 3 Results and Discussion

### 3.1 Structural and Textural Properties

The seeding-free synthesis of zeolite Y catalysts with a layer-like morphology was performed by adding an organosilane as growth modifier and thus creating a hierarchically ordered morphology. The synthesis was aimed to be as straightforward as possible, thus without a seeding step. The lowering of the synthesis gel basicity by adding sulfuric acid was accordingly applied.^4^ This concept was adapted and modified the way that the organosilane TPOAC was added as a growth modifier, the crystallization temperature was reduced from 100 to 75 °C, and PP-bottles were used instead of stainless steel autoclaves for the zeolite synthesis.

**Figure 1:**
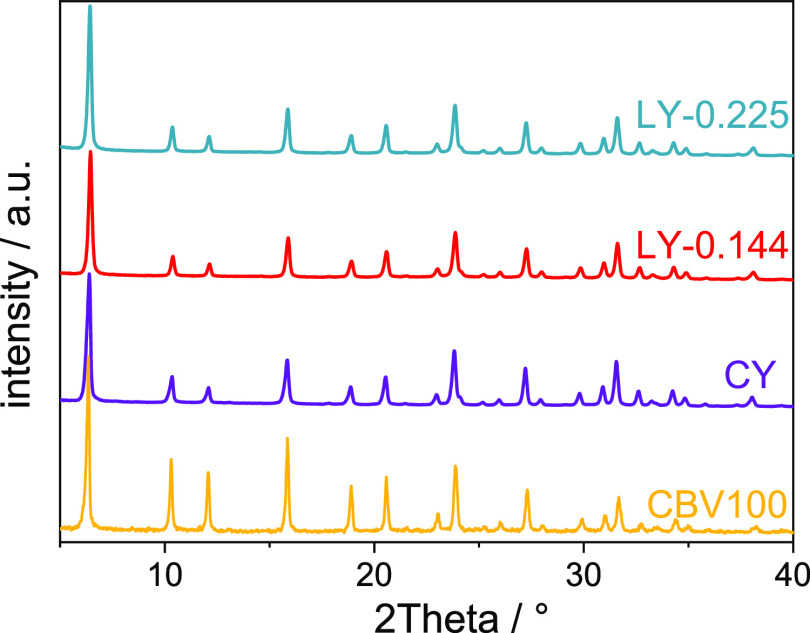
XRD patterns of the layer-like zeolite Y samples (LY-0.225, LY-0.144) and-for comparison-of the conventional zeolite Y sample (CY) and a commercial CBV100 zeolite.

**Figure 2:**
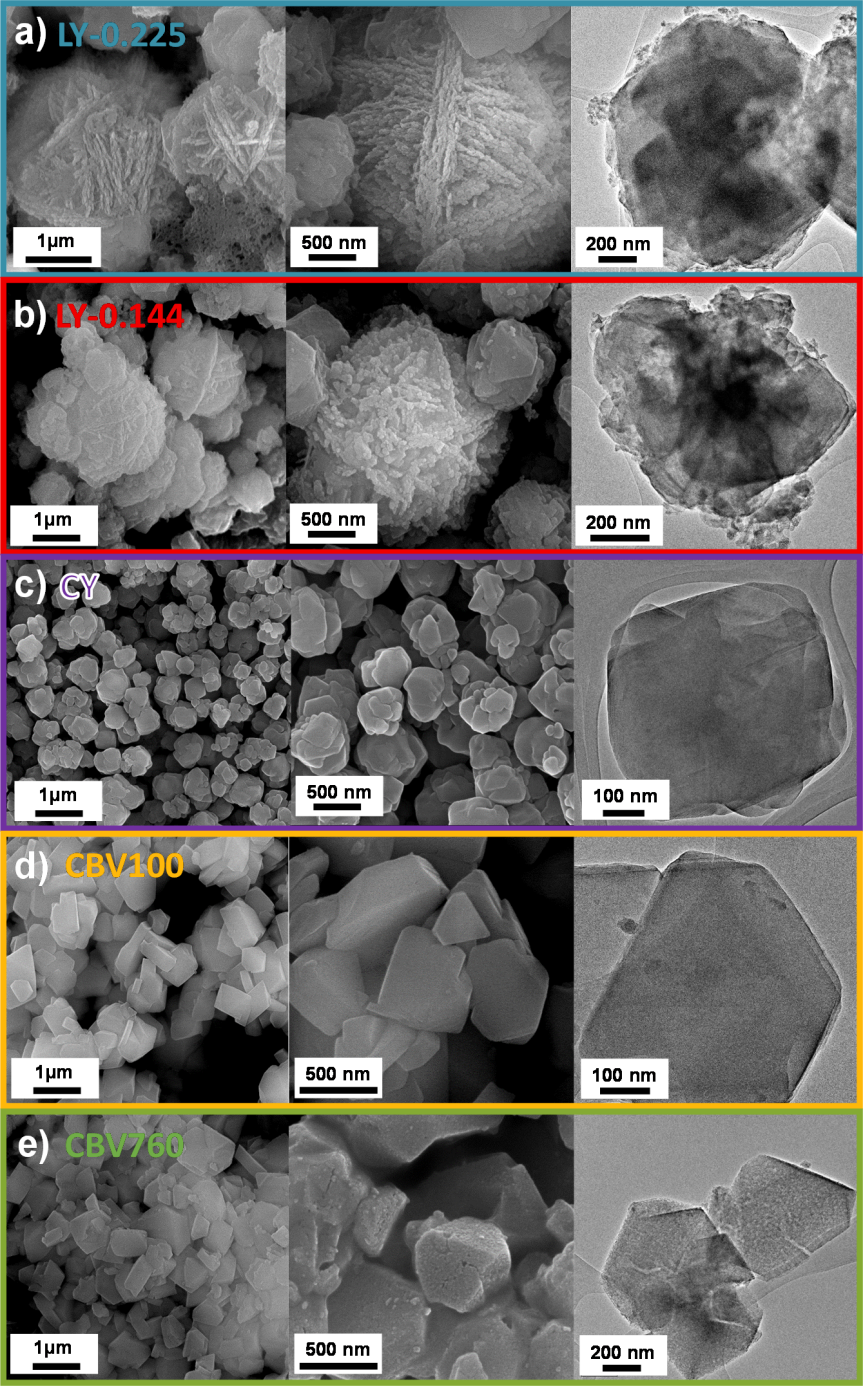
SEM and TEM images with different magnifications of the zeolite samples LY-0.225 (a), LY-0.144 (b), CY (c), CBV100 (d), and CBV760 (e).

In Figure 1, it can be seen that the zeolite Y samples synthesized in the presence of the organosilane perfectly match the characteristic reflexes of a FAU-type zeolite, here demonstrated with the XRD powder pattern of commercial faujasite with a similar Si/Al ratio (CBV100). No impurities of zeolite P (GIS)^29^ or zeolite A (LTA),^30^ the commonly competing zeolite phases during the synthesis of faujasite zeolites, could be detected. In comparison, the zeolite Y sample (CY) synthesized in the absence of the organosilane shows a similar XRD pattern. The relative crystallinity values of the layer-like zeolite Y samples are comparable and in the range of 87–99% of CY zeolite sample (Table 1). The broadened reflexes or lower intensities are observed on XRD patterns (Table S1). This can be referred to either the higher amount of FAU/EMT intergrowth within the dominant FAU phase^13^ or the smaller primary crystal sizes and thus the hierarchical morphology of the zeolite particles.^31^ The latter is evidenced in SEM studies (Figure 2).

**Table 1:** Chemical Composition, Relative Crystallinities, and Textural Properties Derived from ICP–OES, XRD, and Low-Temperature N_2_-Physisorption, resp., of Studied Zeolites

zeolite sample	Si/Al^a^ (-)	relative cryst. (%)	Na/Al^a^ (-)	*S*_BET_^b^ (m^2^ g^–1^)	*S*_ext_^c^ (m^2^ g^–1^)	*V*_micro_^d^ (cm^2^ g^–1^)	*V*_tot_^e^ (cm^2^ g^–1^)	*V*_meso_^f^ (cm^2^ g^–1^)
LY-0.225	2.83	99	0.78	842	76	0.29	0.44	0.15
LY-0.225-H	3.00		0.18	689	64	0.24	0.40	0.16
LY-0.144	2.73	87	0.78	782	60	0.28	0.39	0.11
LY-0.144-H	2.88		0.14	608	55	0.22	0.33	0.11
CY	2.34	100		966	20	0.36	0.42	0.06
CBV100-H	2.73			890	55	0.32	0.36	0.04
CBV760-H	28.97		0.00	913	313	0.34	0.53	0.19

a Molar ratios derived from the ICP–OES method.

b Specific surface area (BET method).

c Specific external surface area (*t*-plot method).

d Micropore volume (*t*-plot method).

e Total pore volume (single point adsorption at p·p_0_^–1^ = 0.984).

f Mesopore volume (*V*_tot_ −*V*_micro_, “non-micropore volume”).

The effect of adding the organosilane TPOAC to the synthesis gel and thus obtaining zeolite Y particles with a different morphology compared to that of a conventional faujasite (CY) is clearly visible in the SEM images in Figure 2. The layer-like zeolite Y samples contain particles with a plate-like morphology. The majority of these particles form a spherical shape consisting of branched plates with three- and four-folded geometries arranged in the manner of the skeleton of a cuboctahedron (Figure 2a,b) similar to the layer-like zeolite X particles described by Inayat et al.^5^ This morphology provides a hierarchical pore system where the void space between the branched plates is seen as an additional macropore system. A further advantage is the reduction of one dimension to layer-like crystals which causes a significant shortening of the diffusion path length of molecules within the zeolitic framework. The particle sizes are around 1.5 and 2.5 μm for the layer-like samples LY-0.144 and LY-0.225, respectively. The average aggregated cuboctahedron size is increasing proportionally to the TPOAC/Al_2_O_3_ molar ratio of the synthesis gel. Additionally, a minor portion of plate-like particles is arranged in smaller intergrowns and the plates seem to be stacked on top of each other with some rotation between the plates and a more dense core. The particles’ mass share of different sizes is provided in Figure S1. Thus, an additional hierarchical order is given from the smaller crystals having a higher frequency of intergrowth/stacking with each other and thus having more intercrystalline void space. Because the organosilane TPOAC also acts as a mesoporogen, an increased content in the synthesis gel should also lead to an increased number of intracrystalline mesopores.^5,32^ In comparison, the conventional CY synthesized in the absence of TPOAC has only one fraction of particles having an average diameter of 0.60 μm and consisting of larger intergrown primary crystals, which often have the morphology of an octahedron (Figure S1). The zeolite Y sample synthesized in the absence of the organosilane shows a morphology without any layer formation, almost round-shaped edges, and homogeneously appearing crystal phase with well visible parallel-oriented structure frames at higher magnification, indicating a high crystalline, microporous structure (Figure 2). In contrast, the zeolite Y samples synthesized in the presence of the organosilane TPOAC show a significant change in morphology as already described. The edges of the particles, and in particular of the intergrown primary crystals, are sharper and having a layer-like morphology. The particle itself has a more heterogeneous morphology consisting of many intergrown layer-like crystals, which can be seen in detail at a higher magnification. The single layer-like crystals became thinner the higher the TPAOC content in the synthesis was. The layer-like particles are of thickness between 50 and 200 nm. Taking into account that the dimension of a faujasite (zeolite Y) unit cell is about 2.47 nm,^33,34^ the shown plate has a thickness of only about 20–70 unit cells. These examples can give an estimation of how the plate thickness is decreasing along with an increase of the molar TPOAC/Al_2_O_3_ ratio in the synthesis gel. The plate thicknesses for layer-like zeolite X samples from previous studies were in the range of 50–100 nm.^14^ Nevertheless, parallel oriented frames of the faujasite crystal structure are also visible in all layer-like zeolite Y samples at higher magnification and indicate, in accordance with the XRD results, a high crystallinity, which is also confirmed by nitrogen physisorption as the BET specific surface areas of all layer-like zeolite Y samples are just 13–19% lower compared to the conventional zeolite CY sample. The decreased BET specific surface area can be partly caused by the layer-like morphology.^5,6^ For comparison purposes, the SEM and transmission electron microscopy (TEM) micrographs of commercial CBV100 and CBV760-H samples were presented. The dealuminated CBV760-H zeolite displayed a secondary mesopore system well-seen on SEM and TEM micrographs (Figure 2).

The additional pore systems in the range of macro- and mesopores are also assumed to be present in the layer-like zeolite Y samples according to the interpretation of the electron microscopic images and literature; this is also confirmed by nitrogen physisorption data (Table 1) and pore size distributions (Figure S2). The mesopore volumes for the layer-like samples derived from it are 0.11 cm^3^·g^–1^ for LY-0.144 having the lower TPOAC/Al_2_O_3_ ratio and 0.16 cm^3^·g^–1^ for LY-0.225 of the higher TPOAC/Al_2_O_3_ ratio. In addition, the heterogeneous structure of the particles in layer-like zeolites seen on TEM images, compared to the homogeneously appearing structure of the CY sample is a confirmation of the mesoporogen effect of TPOAC. Along with the increased mesopore volume also the external surface area is increased with a layer-like morphology occurrence.

One of the most important findings is that the described layer-like zeolite samples are confirmed to be zeolite Y having a Si/Al molar ratio higher than 1.5, as this is the first described synthesis of layer-like zeolite Y in the presence of an organosilane and without using seeding. With Si/Al molar ratios between 2.73 and 2.83, they can be considered as high-silica zeolite Y materials. The presence of the organosilane TPOAC in the synthesis gel also leads to layer-like zeolite Y with Si/Al molar ratios higher than 2.34 of the zeolite Y (CY) synthesized in the absence of TPOAC. This tendency of a higher Si/Al molar ratio using an organosilane was already seen in a study from Tempelman et al.^18^

When an ion-exchange is carried out to obtain the NH_4_-form followed by a calcination step to obtain the H-form, a slight change in textural properties of layer-like zeolites is detected. The total pore volume and the micropore volume are decreasing, while the mesopore volume stays constant. The values for the specific surface areas, BET, and external are also decreasing. The loss in pore volume and specific surface area can be probably related to a partial leaching of framework atoms and thus creating defects during the ion-exchange process. It should be mentioned here that it was not possible to transfer the CY zeolite sample into the H-form. The structure collapsed, which is considered to be the result of a too low Si/Al molar ratio, namely, 2.34. This was also seen in the study of Liu et al.^4^ Therefore, we chose the commercially available zeolite Y samples CBV100-H (after NH_4_-exchange and calcination) and CBV760-H (purchased in H-form) as the reference materials in the catalytic cracking of LDPE as the test reaction.

### 3.2 Acidity Studies—Nature and Accessibility of Acid Sites

**Figure 3:**
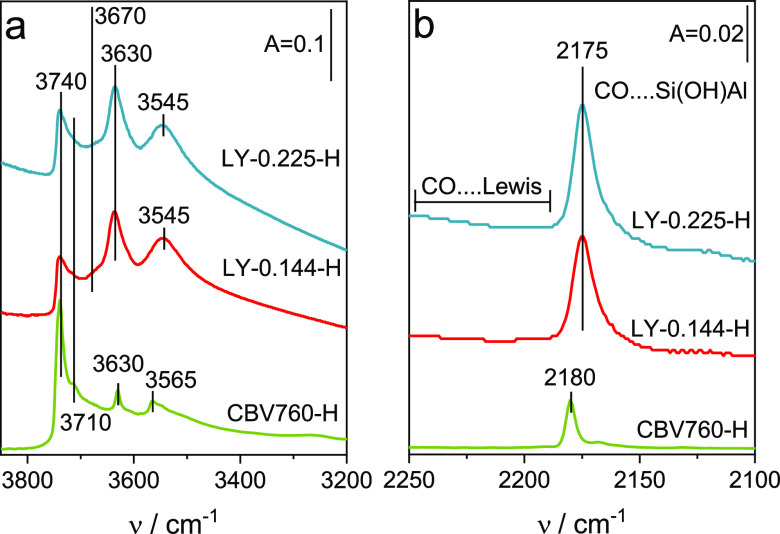
FT-IR spectra of OH groups (a) and the CO interacting with surface acid sites (b) in the materials studied.

The detailed characteristic of Brønsted and Lewis acid sites was derived from the FT-IR studies (Table 2). Starting from Al molar concentrations of 3106–3299 μmol·g^–1^ for the layer-like LY-0.144-H and LY-0.225-H, only 50% of the Al atoms are detected by NH_3_ sorption (Table 2, B + L—sum of Brønsted and Lewis sites). This divergence can be assigned both to the presence of Na^+^ cations (Table 1) and nonacidic aluminols Al–OH. Indeed, the band at 3670 cm^–1^ (Figure 3a) confirms the share of nonacidic Al atoms in the layer-like materials. The nonacidic aluminum species are rather formed in the course of the synthesis protocol than during ion-exchange or calcination procedure. This is confirmed by only slight changes of Si/Al ratios after ion-exchange and calcination procedures.

**Table 2:** Acidity Characteristic Derived from NH_3_, CO, and di-TBPy Adsorption FT-IR Studies

					B	L		
zeolite sample	Al^a^ (μmol·g^–1^)	B^b^ (μmol·g^–1^)	L^b^ (μmol·g^–1^)	B + L (μmol·g^–1^)	NH_3_^350^/NH_3_^200^^b^ [-]	NH_3_^350^/NH_3_^200^^b^ [-]	Δν_CO···OH_^c^ (cm^–1^)	AF_B_^d^ (%)
LY-0.225-H	3106	991	603	1594	0.53	0.88	277	61
LY-0.144-H	3299	1029	582	1611	0.31	0.91	266	11
CBV100-H	3128	3085	35	3120	0.55	0.30	275	2
CBV760-H	493	335	85	420	0.85	0.60	354	51

a Concentration of Al from the ICP–OES method.

b Data derived from NH_3_ adsorption IR studies: the concentration of Brønsted (B) and Lewis (L) acid sites, and the acid strength of sites (NH_3_^350^/NH_3_^200^).

c Strength of the Si(OH)Al groups determined from low-temperature CO sorption of IR experiments.

d Accessibility factor calculated as the share of Brønsted acid sites accessible for di-TBPy of the number of the sites able to react with ammonia (c_di-TBPyH^+^_/c_NH_4_^+^_).

Regardless of the amount of TPOAC, the number of Lewis and Brønsted acid sites in H-forms is similar. Comparable intensities of the Si(OH)Al group bands at 3545 cm^–1^ and 3630 cm^–1^ (Figure 3a) confirm the similar concentration of Brønsted acid sites in layer-like materials. Among the layer-like samples, only LY-0.225-H material offers the Si(OH)Al hydroxyls of similar strength to bulk commercial CBV100-H. Then, the conventional CBV100-H compared to the dealuminated CBV760-H obviously has the protonic sites of significantly lower strength. This is seen in the ammonia thermodesorption data (NH_3_^350^/NH_3_^200^) and the value of Δν_CO···OH_, representing the downshift of the Si(OH)Al group band after hydrogen bonding with CO molecules (Table 2). Still however, the strength of acid sites is the parameter differentiating among organosilane-derived samples: the protonic sites of the highest strength are located in the material synthesized with the higher content of organosilane. Therefore, it can be assumed that the presence of TPOAC either facilitates the location of Al atoms in T-positions offering the most acidic Si(OH)Al hydroxyls or prevents the extraction of Al atoms from these positions. The introduction of TPOAC in the synthesis protocol resulted also in the presence of the Lewis acid sites of higher strength than in commercial CBV100-H and CBV760-H.

What distinguishes the layer-like zeolite Y samples from the commercial CBV100-H sample is the significantly higher amount of Lewis acid sites: LY: 513–703 μmol·g^–1^; CBV100-H: 35 μmolg^–1^. For the assessment of the Lewis sites’ nature, carbon monoxide molecule was employed as a probe (Figure 3b—CO···Lewis). The spectra of CO interacting with the surface sites in organosilane-derived zeolites show only the band at 2175 cm^–1^ identifying the CO interaction with Brønsted acid sites as the only species. There are no bands visible in the 2250–2180 cm^–1^ frequency region that could allow concluding on the presence of CO interaction with Lewis acid sites. The Lewis acid sites located in the layer-like samples are therefore of low strength as they are not able to bind weakly basic CO molecules, in contrast to highly basic ammonia molecules (Table 2). Such Lewis acid sites can be a part of extra-framework aluminum species.

Information on the accessibility of protonic sites in studied layer-like materials was obtained from di-TBPy adsorption (Figure S3). The bulky di-TBPy cannot enter the micropores, even in wide-pore zeolites therefore this molecule is widely used to probe the Brønsted sites exposed on the external surface. The maximum intensity of the 1615 cm^–1^ diagnostic band attributed to di-TBPyH^+^ cations and its absorption coefficient served to calculate the concentration of protonic sites able to interact with the probe (Table 2). The latter values were referred to the concentration of acid sites determined from the adsorption of ammonia and presented as the accessibility factor values (AF_B_, Table 2). Nearly no sites in nonmesoporous zeolite CBV100-H were available (AF = 2%), while in commercial CBV760-H, more than half of protonic sites were able to protonate di-TBPy (AF = 51%). Importantly, the layer-like zeolite LY-0.225-H offers the sites very easily reachable to di-TBPy, and the AF_B_ factor of LY-0.225-H exceeds the one found for super dealuminated ultrastabilized zeolite CBV760-H. Keeping in mind that the protonic site density in layer-like zeolites is above threefold higher than in commercial CBV760-H, the enhanced accessibility of Brønsted sites can importantly benefit the cracking performance over these mesostructured materials.

The presence of hierarchical porosity is also beneficial to an increased population of the silanol groups (Figure 3a). Isolated silanols (the band at around 3740 cm^–1^) are increasing in amount along with the TPOAC content. The increase of isolated Si–OH groups is in accordance with an increase in external surface area of layer-like zeolites Y and the commercial CBV760-H. Besides, isolated single (SiO)_3_Si–OH groups oscillating at 3740 cm^–1^, the bands of internal silanols at 3710 cm^–1^, are also distinguishable in the FT-IR spectra presented in Figure 3a. While the external silanols are needed to the crystal lattice termination, the internal ones result from unbalanced charges in the zeolitic framework. The higher relative population of latter species supports our conclusion on the existence of some defects in micropores due to non-framework Al-atoms embedded in organosilane-derived layer-like zeolites Y. Indeed, the silanol defects are an intrinsic feature of layered zeolites because of the high ratio of the surface to bulk. Based on the ^29^Si MAS NMR studies, it has been proved that the charged organic structure directing agents are responsible for the occurrence of defects in zeolites.^35^ Hydrophilic silanol groups in crystalline aluminosilicates (zeolites) or silicates are characterized by a weak or moderate acid strength; therefore, in addition to bridging hydroxyls Si(OH)Al, they can contribute to some reactions. The internal H-bonded silanols were found to be more active and more selective compared to external silanols in the Beckmann rearrangement.^36^ The silanols in ferrierite and ZSM-5 were proven to react with C_8_-olefins as proton donors, contrary to silanols in amorphous silica. Accordingly, the role of both external and intrinsic silanols cannot be neglected in the catalytic cracking of polymers and their secondary transformations especially in the zeolitic structures were their abundance is significant.

To sum up, among the TPOAC-derived mesostructured materials, LY-0.225-H offers the optimal textural properties and acidic function defined as the number, strength, and accessibility of protonic sites.

### 3.3 Layer-like Zeolite Y in Catalytic LDPE Cracking: Thermogravimetric and *Operando* FT-IR Studies

**Figure 4:**
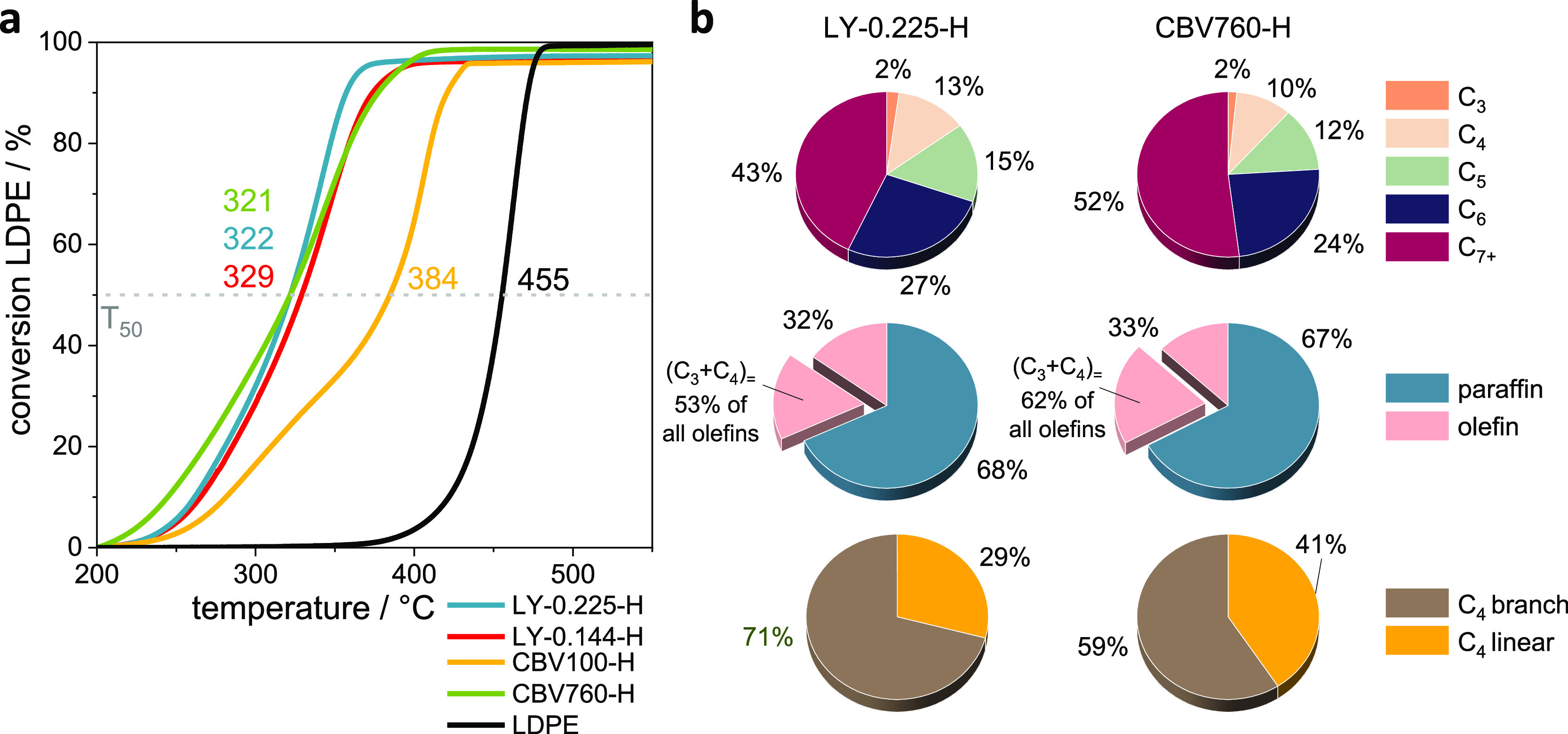
(a) Conversion in a LDPE-cracking reaction for layer-like zeolite Y samples (LY-0.144-H and LY-0.225-H) and commercial zeolite Y samples (CBV100-H and CBV760-H) as catalysts and the conversion in the absence of any catalyst (LDPE). (b) Distribution of the cracking products: selectivity (%) (upper); the share (%) of the paraffin and olefin fractions (middle); and the ratio between the branched and linear compounds in the C_4_ fraction (lower).

To bring the achieved textural and morphological properties in the context of the catalytic activity of these layer-like zeolite Y materials, the catalytic cracking of LDPE was chosen as the test reaction. As already mentioned above, the commercial zeolite Y samples CBV100-H and CBV760-H were taken as reference materials. The results of the LDPE catalytic cracking over the layer-like and the commercial faujasite zeolites are given in Figure 4, where the conversion is plotted against the reaction temperature (Figure 4a). The temperature at 50% conversion is denoted as *T*_50_. For additional comparison, the thermal cracking of LDPE was also performed (curve denoted as LDPE). In this study, at lower temperatures, up to 310 °C, the commercial sample CBV760-H shows the best catalytic performance. In terms of measurement accuracy, the layer-like sample LY-0.225-H showed only slightly inferior performance at this point. Having a look at the region of conversions higher than 50%, LY-0.225-H shows a superior catalytic activity (*T*_80_ = 347 °C) over CBV760-H (*T*_80_ = 359 °C). It must be noted, that the layer-like zeolite LY-0.225-H sample showed a catalytic performance comparable with that of the super dealuminated ultrastabilized zeolite CBV760-H. It should be highlighted here that the synthesis and general preparation of this highly catalytically active layer-like zeolites Y were the bottom-up routes kept very simple compared to the synthesis and postsynthesis treatments necessary for obtaining the CBV760-H zeolite. The additional postsynthetic treatments of SDUSY zeolite include steaming and acid-leaching to achieve a hierarchical pore structure and an increased Si/Al molar ratio to provide a high catalytic activity.

Two the most active materials, that is, commercial CBV760-H and layer-like LY-0.225-H, were also investigated under *operando* conditions in a FT-IR cell with GC–MS detection for the assessment of catalyst selectivity. Wide-pore zeolites, that is, mordenite,^37^ β,^38 −40^ and mesoporous ultrastabilized zeolite USY,^41,42^ tested in the catalytic degradation of polyethylene were reported to produce the higher amount of liquid fraction compared to medium-pore zeolite ZSM-5. The LDPE cracking over ZSM-5 zeolite favored the higher amounts of gases formed by secondary recracking reactions. The hydrocarbon products formed over USY were predominantly alkanes with less share of alkenes and aromatics, in line with the reported predominance of hydride-transfer (HT) processes^43^ which increases the selectivity toward paraffin in wide-pore zeolites. The quantitative analysis of the overall reaction products by GC–MS shows that in comparison with super dealuminated ultrastabilized zeolite CBV760-H, the layer-like material LY-0.225-H yielded more in value-added C_3_–C_4_ gases and C_5_–C_6_ liquid fraction with a significant decrease in the amount of C_7+._ This higher cracking efficiency can be ascribed to the high amount of easily accessible Brønsted sites (Table 2). The strength of protonic sites seems to be the secondary importance; the 3.5-fold higher amount of Brønsted sites in LY-0.225-H than in commercial material CBV-760-H is more beneficial for cracking competence than sites of high strength but rarely populated (Table 2). High abundance of the internal silanols in layer-like zeolite LY-0.225-H is also important. The concentration of moderate acid strength of silanol proton donors, in addition to bridging hydroxyls Si(OH)Al, allows us to retain the adsorbate molecule on the catalyst surface long enough to benefit the cracking efficiency. This conclusion is supported by our earlier studies reporting the high polypropylene cracking efficiency of Brønsted acid sites dispersed in mesoporous aluminosilicate HAlMCM-48.^44^ Highly developed external surface area and excessively populated and accessible Brønsted sites of medium strength in LY-0.225-H provide nonconstrained polymer–acid site interactions which place this layer-like material among the suitable catalysts for LDPE cracking. Furthermore, the re-cracking efficiency would have been significantly inhibited in comparison to the nonhierarchical CBV100-H material.

The HT ability of the cracking catalysts can be measured with the paraffin/olefin ratio. The HT processes, such as bimolecular reactions, are facilitated in the presence of the catalysts characterized by a large number of the acid site and with bigger size of internal cavities. Both zeolites, the commercial and layer-like one, are however characterized by similar paraffin/olefin ratios (Figure 4b). The type of pore hierarchy (intracrystalline mesoporosity in CBV760-H vs intercrystalline mesoporosity in LY-0.225-H) is therefore irrelevant for the olefin share in the final product (Figure 4) in spite of some diversity in mesopore volumes in favor of commercial zeolite. Furthermore, high number of accessible protonic sites (Table 2) should facilitate the formation of olefin in layer-like zeolite because the bimolecular HT reaction needs adjacent sites to occur. The higher acid strength enhances also the higher turnover numbers and the less hydrogen transfer reactions take place. In addition to the Brønsted acid sites, Lewis sites are believed to initiate the paraffin cracking and participate in secondary HT processes^45^ as a carbenium ion is formed by the abstraction of a hydride ion from a saturated hydrocarbon by the electron acceptor site. Accordingly, in LY-0.225-H with high abundance of Lewis acid sites, the HT reactions should be also favored. A similar HT activity commercial bulk and layer-like zeolites can therefore result from mutually compensating effects: more developed external surface area and the presence of protonic sites of high strength in CBV760-H and high density of medium-weak protonic sites in LY-0.225-H. Still however, in layer-like zeolite with a higher cracking efficiency, the significant olefin abundance in the (C_3_ + C_4_)_=_ fraction is observed. It suggests that the production of low olefins takes place in the microporous environment of layer-like zeolite LY-0.225-H which is not affected by additional mesoporous characteristics such as the interior mesopore system in CBV760-H. Similarly, in layer-like LY-0.225-H due to steric constraints resulting from less spacious internal voids (Table 1) than in the commercial CBV760-H also the rate of isomerization is enhanced. The less spacious internal voids LY-0.225-H (Table 2) also improve the rate of isomerization in the C_4_ fraction. In contrast, a highly developed intracrystalline secondary mesopore system in commercial CBV760-H, by reducing of the contact time of the reagents, facilitates the higher production of *n*-olefins by preventing the carbocations from a skeletal isomerization before their desorption as iso-olefins.

The course of conversion curve slopes will find also reflection in the evolution of the various nature of carbonaceous deposit in a layer-like material and its bulk analogue. Up to 45% conversion, a commercial material CBV760-H is more efficient in LDPE cracking due to the high strength of Brønsted sites, thus the higher the turnover numbers. However, at a higher temperature range (above 320 °C), the protonic sites in CBV760-H were partially poisoned by coking and the LY-0.225-H catalyst became more active. The high-density medium strength protonic sites in LY-0.225-H benefit to the catalytic performance. High abundance of the Brønsted acid sites in LY-0.225-H provide the conditions in which a large part of protonic sites is still catalytically active despite the undeniable fact that a significant part of them was eliminated by coking. Still, the layer-like LY-0.225-H is an example of the defect-enriched catalyst whose cracking catalytic performance is improved by the presence of acidic silanols, but the lifetime might be shortened by undesirable coke production.

### 3.4 Coke Analysis and *Operando* IR-MS Studies of Coke Burn-Off

**Figure 5:**
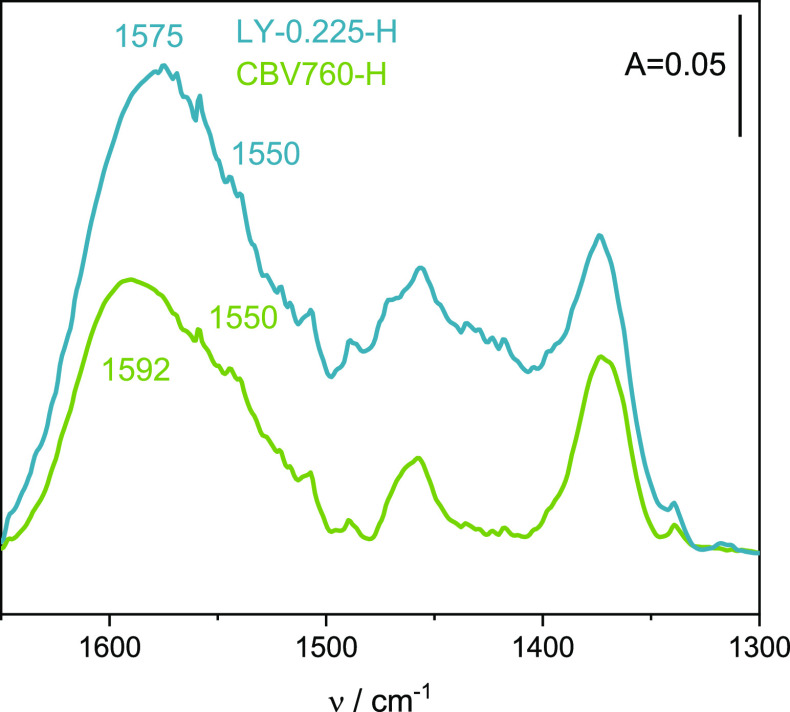
FT-IR spectral characteristics of coke residues in CBV-760-H and layer-like LY-0.225-H catalysts.

Catalyst deactivation by pore blockage (fouling) is very prevalent in polymer catalytic cracking.^44^ The coking phenomenon involves many secondary successive reactions, mainly bimolecular and condensation and hydrogen transfer processes. The deactivation of the catalyst can change its activity and selectivity. The coke species formed during the LDPE cracking over two of the most active catalysts, that is, commercial CBV760-H and layer-like LY-0.225-H (Figure 5), can be identified by the complex band around 1650–1500 cm^–1^. In the bulk CBV760-H catalyst, the coke IR band is complex and located at higher frequencies 1592 and 1550 cm^–1^ when compared to layer-like LY-0.225-H in which coke residues are more homogeneous as characterized by a much narrower band at 1575 cm^–1^. The species formed in LY-0.225-H with the protonic sites of medium-low strength (Table 2) consists of mainly conjugated olefinic species as coke forming compounds. Strong acid sites in CBV760-H facilitated oligomerization and condensation reactions to produce C_6_H_7_^+^ olefinic cation (CH_2_=CH–CH]CH=CH]CH^+^),^46^ cyclic alkenyl carbenium ions C_6_H_9_^+^, polymethyl-benzenes (e.g., 1,2,4-trimethylbenzene, 1,2,3-trimethylbenzene), and also hydrogen-deficient polyaromatic species^47^ identified by the 1592 cm^–1^ band. The 1550 cm^–1^ band can be ascribed to C=C ring vibrations in polynuclear aromatics (e.g., anthracenes and naphthalenes).^48,49^ The amount of coke residues estimated from TGA was found the be higher for layer-like zeolite with intercrystalline mesoporosity (15%) than for bulk commercial analogue with intracrystalline mesopore CBV760-H (7%). The acid sites of high strength imply faster chemical steps and stronger retention of coke molecules and precursors. In our case, however, a higher density of easily accessible acid sites in LY-0.225-H led reactant molecules to entangle in successive steps along the diffusion path within zeolite Y crystallites, promoting secondary bimolecular processes, that is, oligomerization and condensation reactions. Because the former provides higher diffusional restrictions due to lower values of micropore volumes (Table 2), the carbonaceous species are favorably produced on its external surface. The FT-IR characteristics inform, however, that the coke species in layer-like zeolite Y is more aliphatic, while hydrogen-deficient polyaromatic species are present in CBV760-H.

Despite the benefits to the catalytic cracking performance due to moderate acid strength of silanols, their presence brought also issues with faster coking of the zeolite catalyst. Coke species, that is, heavy hydrocarbons, are easily attached to the silanol-enriched surface. The higher the silanol population, the more negative the coking effects are. The different type of silanols, external versus internal, showed various activities toward coke deposit formation.^50^ The higher amount of coke was found in zeolite LY-0.225-H with a higher amount of internal silanols, while zeolite CBV760-H possessing the external silanol groups was deactivated at a lower extent. It points to involving the more acidic silanols into coke species formations. Still however, due to structure constraints, its character is more olefinic than in commercial zeolite CBV760-H. This retention of olefinic coke precursors in LY-0.225-H occurs either because the cracking products can be also accumulated in the micropores of zeolite or cannot be effectively eliminated from the microporous environment due to a steric hindrance.

**Figure 6:**
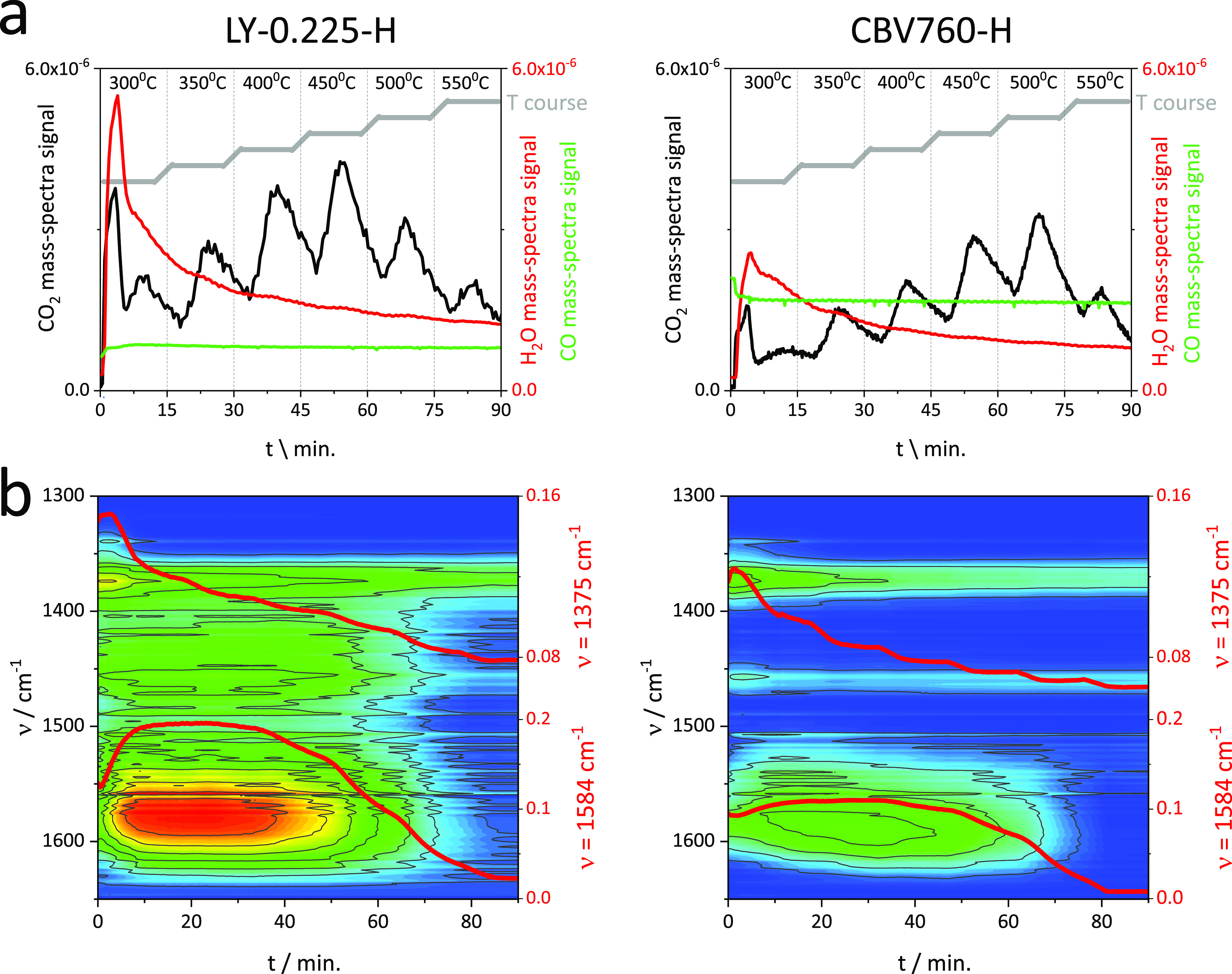
(a) Mass spectrum signal of CO_2_ (black line), CO (green line), and H_2_O (red line) and (b) 2D maps of FT-IR spectra with traces of 1375 and 1584 cm^–1^ bands (red lines) during TPO experiments. The temperature course (*T* course) in TPO experiments is indicated in the upper part of the graph.

A regeneration process involving coke combustion is generally employed for assessing the more detailed information of coke nature. This was derived from monitoring CO_2_, H_2_O, and −CH_3_ and C=C species formed during the thermoprogrammed oxidation (TPO, 5% of O_2_ in N_2_) of carbonaceous moieties tracked by mass spectrometry (Figure 6a) and FT-IR spectroscopy (Figure 6b). The TPO-IR measurements were performed at several temperatures (300, 350, 400, 450, 500, and 550 °C). At each annealing stage, the temperature was kept for 10 min and then increased with a rate of 10 °C/min.

The structure of the TPO profiles provided information on the diverse speciation of coke deposit; therefore, its combustion pathway can be addressed to the acidic and textural properties of the bulk and layer-like zeolites Y studied. The combustion of the coke deposit located on the external surface requires lower temperatures than the burning of coke inside the pores. Indeed, the apparent activation energy for the oxidation of coke species formed inside the pores has been reported to be half the intrinsic activation energy or the activation energy of the combustion of coke located on the external surface.^44,51^ Evolution of CO or CO_2_ during the TPO has been found to be dependent on the oxidation mechanism strongly related with the coke morphology.^52^ The oxidation of the coke molecules is initiated over their hydrogen atoms with the formation of oxygenated intermediates decomposed subsequently into CO and CO_2_. High carbon species are burnt off, therefore, at higher temperatures. The proposed mechanism involves a free carbon site C_f_ available for oxygen chemisorption and −C(O) and −C(O_2_) dissociated and undissociated surface oxide species releasing finally CO and CO_2_.
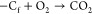
1
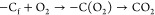
2
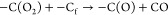
3
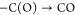
4

Accordingly, the first moiety observed in the TPO profile is water from light coke dehydrogenation, followed by CO and CO_2_ production by heavier species oxidation (Figure 6a). However, in presented TPO profiles, the intensive production of water (*m*/*z* = 18) at temperature 300 °C accompanied by the sharp CO_2_ signal was ascribed to the burning of noncracked LDPE surrounding the outer surface of the catalysts grains as the conversion under isothermal (220 °C) *operando* conditions does not reach 100% for both LY-0.225-H and CBV760-H. At a higher annealing temperature, that is, 350 °C, the extent of water and CO_2_ production is smaller and spread over time. The significantly higher relative ratio of H_2_O/CO_2_ for CBV760-H indicates still an important share of the combustion of the LDPE, most probably located in intracrystalline mesopores, while CO_2_-enriched combustion stream released from the layer-like LY-0.225-H suggests the starting of the carbonaceous deposit oxidation. The differences in the hydrocarbon-derived deposit oxidation are also detectable in the course of the 1375 cm^–1^ (corresponding to the bending vibrations of C–H in paraffinic and unsaturated compounds) and 1590–1550 cm^–1^ bands (corresponding to the stretching C=C vibrations), which were chosen as the indicators of coke presence in FT-IR spectra recorded during the catalyst regenerations (Figure 6b). For both fouled materials, the highest drop the CH_3_– band (the 1375 cm^–1^) starts at 300 °C, which confirms again the oxidation of LDPE residues. In spite of a higher amount of the coke species in the layer-like material, what is manifested as the twofold higher intensity of the C=C band at 1590–1550 cm^–1^ than in commercial zeolite, its combustion is more progressed and 80% of the coke is oxidized before reaching 500 °C. It is due to the higher abundance of the external surface olefinic species in a layer-like material than in a commercial one, as documented by the lower temperature CO_2_ signal evolution. At temperatures as high as 500 °C, the combustion of coke in CBV760-H is correlated with the higher production of CO_2_ (the relative ratio of CO_2_/H_2_O is higher for CBV760-H than for LY-0.225-H), providing an evidence for the oxidation of hydrogen-deficient polyaromatic coke species populated extensively in commercial zeolite. The presence of the strong acid sites in CBV760-H facilitates the oligomerization processes and the slower diffusion of basic intermediates and finally the coke species formation is faster. The regeneration of the catalyst CBV760-H in more severe conditions could be assigned tentatively to the coke species being trapped inside the zeolite grains. The commercial catalyst CBV760-H is characterized by a more mesoporous structure (Table 1); therefore, a high temperature of CO_2_ production cannot characterize internal coke but external species of polyaromatic nature. As mentioned, high population of silanols in both zeolites enhanced coke species retention. On the other hand, besides coke speciation and its location, the catalyst morphology and textural properties were proposed also to influence coke combustion. These features govern the diffusion of oxygen within catalyst pores and thus the variable coke accessibility to oxygen. Accordingly, the coke species oxidation is often qualified as a shape-selective process.^53^ The carbonaceous deposit of less condensate nature was oxidized at higher temperatures on narrow- and medium-pore zeolites than on wide-pore structures. Further studies have showed however that the pore structure is not the main determinant for the rate of coke oxidation on the zeolites but the density of the acid sites.^54^ The greater the number of protonic sites is, the lower the share of coke which requires temperatures above 450 °C to be oxidized. Accordingly, the large quantity of Brønsted acid sites in LY-0.225-H benefits the faster oxidation of coke species despite higher abundance of the latter both in micropores and on external surface.

Intercrystalline macro- and mesopores and interconnectivity between the channels offer a better circulation of oxygen, subsequently influencing positively the contact between oxygen and coke deposits located over the inner surface. In spite of the oxygen rich conditions, the CO production is observed and remains constant throughout the experiment, proving that the steps involved in CO production, (3) and (4), are still decisive due to the presence of the diffusion constraints for the oxygen and the oxidation products in both zeolites tested. Carbon monoxide formed in the oxidation of the coke located on the external surface is rapidly transformed into CO_2_ because the reaction takes place under oxygen-rich conditions. Therefore, the possible reaction mechanism responsible for the CO appearance can be ascribed to the reaction of CO_2_ with the carbonaceous deposit located in the internal or subsurface part of crystals. The restricted diffusion of CO_2_ through the crystal and finally through the coke layer blocking micropore entrances can be responsible for the secondary reaction between the coke layer and CO_2_ giving CO.^55^ Consequently, the CO signal signifies the oxidation of the coke placed in internal crystal environment. Therefore, finally it can be concluded that coke combustion is facilitated with the catalyst LY-0.225-H with high acid site density and the intercrystalline mesoporosity despite the fact that the high population of internal silanols enhanced coke species retention.

## 4 Conclusions

In this study, a seeding-free synthesis of high-silica layer-like zeolite Y samples in the presence of an organosilane (TPOAC) could be presented for the first time. The bottom-up route by using an organosilane acting as the growth modifier as well as mesoporogen was combined with the elegant way of avoiding a seeding-step by reducing the alkalinity of the synthesis gel by adding sulfuric acid. The obtained zeolite Y samples provide large almost spherical particles consisting of intergrown/branched plates arranged in the manner of the skeleton of a cuboctahedron and a minor portion of plate-like particles stacked on top of each other with some rotation between the plates and a more dense core. The layer-like zeolite Y samples have Si/Al molar ratios in the range of 2.73–2.83, high BET surface areas of 782–842 m^2^ g^–1^, and high mesopore volumes of 0.11–0.16 cm^3^ g^–1^. Among the layer-like zeolite Y samples, the sample LY-0.225-H, whose synthesis gel had a TPOAC/Al_2_O_3_ molar ratio of 0.225, showed the best catalytic performance in cracking of LDPE. Its catalytic activity was comparable to the commercial super dealuminated ultrastabilized sample CBV760-H at 50% conversion of LDPE and even exceeds this sample at higher conversions. The layer-like LY-0.225-H zeolite shows the highest concentration of easily accessible Brønsted acid sites, the highest BET surface area, and micropore and mesopore volume. There are many factors having a positive influence on the catalytic behavior in the cracking of LDPE as a test reaction. The layer-like LY-0.225-H zeolite yielded more value-added C_3_–C_4_ gases and C_5_–C_6_ liquid fraction and this was ascribed to the high amount of easily accessible Brønsted sites. The strength of protonic sites was of secondary importance. Despite the fact that the high population of internal silanols enhanced coke species retention on the LY-0.225-H zeolite, its combustion was facilitated. The modified bottom-up synthesis route to obtain high-silica layer-like zeolite Y samples is a promising route to tailor a highly active zeolite catalyst for the upcycling of LDPE by catalytic cracking. It provides an alternative additional seeding-step during synthesis and postsynthetic treatments with acid and steam to create a hierarchical morphology.

## Supporting Information Available

Full width at half-maximum values of x-ray diffraction reflection lines; particle distribution according to their mass share; low-temperature N_2_ sorption isotherms and pore size distribution for studied zeolites; and FT-IR spectra of 2,6-di-tert-butylpyridine (PDF)
