# Supplementary material for: High-Silica Layer-like Zeolites Y from Seeding-Free Synthesis and Their Catalytic Performance in Low-Density Polyethylene Cracking
Source: ACS Appl Mater Interfaces. 2022 Jan 25;14(5):6667–79. doi: 10.1021/acsami.1c21471 (PMC8832398; doi:10.1021/acsami.1c21471)
Supplement: Supplementary file 1 — am1c21471_si_001.pdf [file am1c21471_si_001.pdf]

Supplementary Information

for

High-Silica Layer-like Zeolites Y from Seeding-Free Synthesis and Their Catalytic Performance in Low-Density Polyethylene Cracking

Bastian Reiprich, Karolina A. Tarach*, Kamila Pyra, Gabriela Grzybek, Kinga Góra-Marek

Faculty of Chemistry, Jagiellonian University in Kraków, Gronostajowa 2, 30-387 Kraków, Poland

Karolina Tarach: karolina.tarach@uj.edu.pl

Table.SI 1 Full Width at Half-Maximum (FWHM) Values of X-ray Diffraction Reflection Lines

zeolite sample	(hkl)	FWHM	(hkl)	FWHM	(hkl)	FWHM	(hkl)	FWHM
LY-0.225	(333)	0.204	(440)	0.198	(533)	0.190	(642)	0.200
LY-0.144		0.211		0.207		0.210		0.203
CY		0.197		0.198		0.211		0.189

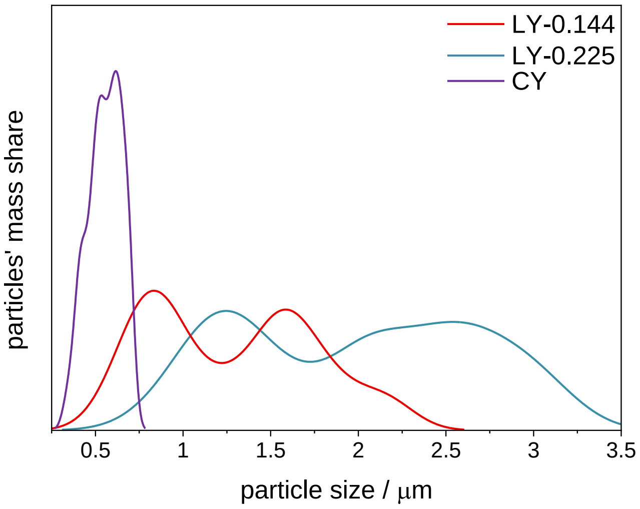

Figure.SI 1 Particles distribution according to their mass share of the layer-like zeolite Y samples LY-0.144 and LY-0.225 and for comparison of the conventional zeolite Y sample CY. The two particle fractions present in the layer-like samples can be found.

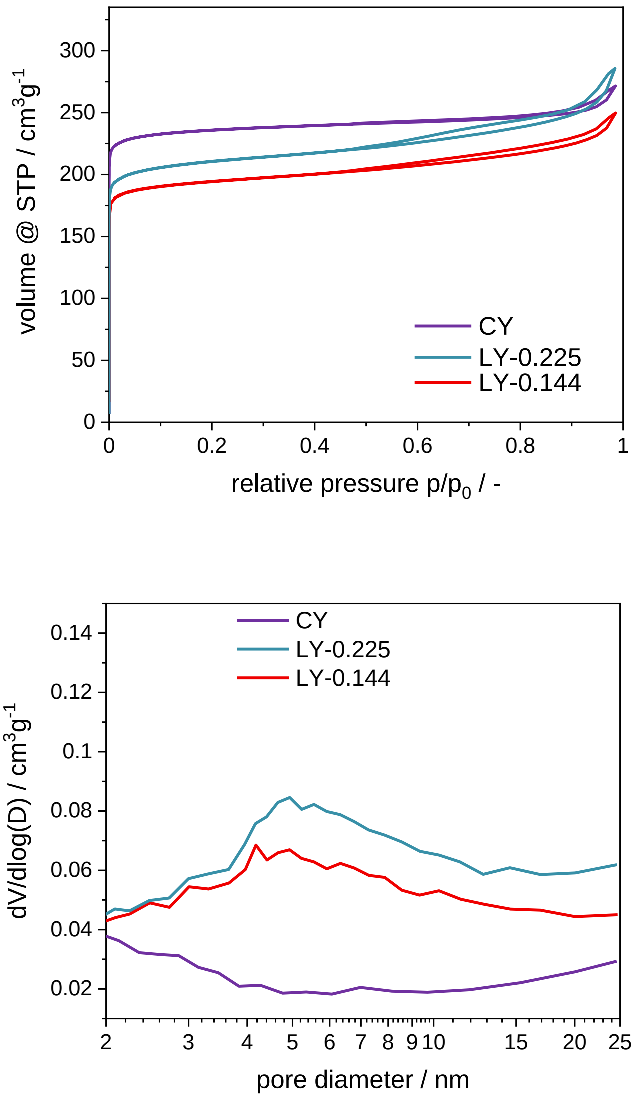

Figure.SI 2 Low temperature N_2 sorption isotherms (upper) and pore size distribution (lower) for studied zeolites.

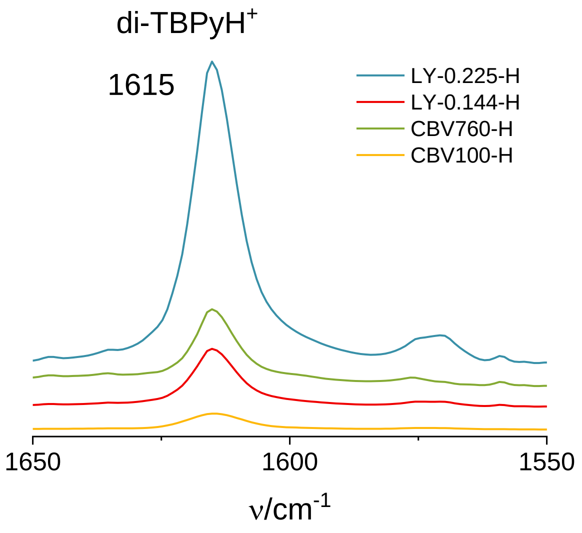

Figure.SI 3 FT-IR spectra of 2,6-di-*tert*-butylpyridine (di-TBPy) adsorbed on the studied materials.
